# *Mycoplasma pneumoniae* pneumonia-associated thromboembolism with plastic bronchitis: a series of five case reports and literature review

**DOI:** 10.1186/s13052-024-01690-1

**Published:** 2024-06-18

**Authors:** Peng Jin, Chunjiao Han, Wei Guo, Yongsheng Xu

**Affiliations:** 1 Department of Respiratory Medicine, Tianjin University Children’s Hospital (Tianjin Children’s Hospital), 238 Longyan Road, Beichen District, Tianjin, 300134 China; 2 Clinical School of Pediatrics, Tianjin Medical University, Tianjin, China

**Keywords:** Mycoplasma pneumoniae, Thrombosis, Plastic bronchitis, Children

## Abstract

**Background:**

*Mycoplasma pneumoniae* pneumonia is a common respiratory infection among children. However, the occurrence of thromboembolism with plastic bronchitis in association with *Mycoplasma pneumoniae* pneumonia is extremely rare. This case series presents five cases of children with *Mycoplasma pneumoniae* pneumonia who developed thromboembolism and plastic bronchitis. The clinical presentation, diagnostic approach, and management strategies are discussed.

**Methods:**

A retrospective analysis was conducted on medical records from a pediatric hospital. Patient demographics, clinical features, laboratory findings, imaging results, treatment modalities, and outcomes were collected.

**Results:**

The patients in our case series presented with varying degrees of respiratory distress, cough, and fever. Imaging studies revealed evidence of thromboembolism based on pulmonary artery occlusion. Bronchial casts were observed by bronchoscopy. Laboratory tests demonstrated elevated D-dimer levels and fibrinogen degradation products. All patients received a combination of low molecular weight heparin anticoagulation and supportive care.

**Conclusion:**

Thromboembolism with plastic bronchitis associated with *Mycoplasma pneumoniae* pneumonia is a rare but potentially serious complication in children. Prompt recognition and management are crucial for improving patient outcomes. This case series highlights the diverse clinical presentations, diagnostic challenges, and treatment strategies for this unique clinical entity. Further research is needed to better understand the pathogenesis and optimal management of this condition.

## Introduction

*M. pneumoniae* is now recognized as a causative agent of respiratory infections across all age groups and a significant contributor to community-acquired pneumonia [1]. In the United States alone, there are over 2 million cases of *Mycoplasma pneumoniae* pneumonia (MPP) reported annually, leading to approximately 100,000 hospitalizations each year [2]. In addition to pneumonia, MPP can also give rise to severe extrapulmonary complications such as pleurisy, hydrothorax, plastic bronchitis (PB), embolism, and multiple organ damage [3]. In this study, we first present a series of cases involving children who experienced thromboembolism and plastic bronchitis associated with *M. pneumoniae* pneumonia. We provide a detailed summary of the clinical findings from five cases demonstrating the presence of embolism and PB in MPP patients.

## Methods

### Patients and definitions

The inclusion criteria were as follows: ① The condition met the diagnostic criteria for MPP: (i) positive results for the serological test (MP-IgM positive and antibody titer ≥ 1:160); (ii) the positive results for MP polymerase chain reaction (PCR) tests of nasopharyngeal secretions. ② The condition met the diagnostic criteria for thromboembolism: CTA/MRA indicates embolism in the blood vessel or heart. ③ The condition met the diagnostic criteria for PB [4]: (i) expectoration of cohesive bronchial casts which assume the shape of the local airways; (ii) bronchial plastic material can be seen in the extracted or coughed up material under bronchoscopy.

The exclusion criteria were as follows: ① Combined with other pathogenic infections. ② Basic diseases, including chronic cardiopulmonary disease, rheumatism, immune deficiency disease, or severe blood system disease. ③ The age was more than 15 years old. All procedures performed in studies were following the Ethics Committee of Tianjin Children’s Hospital.

### Etiological detection

All the children completed the following detection within 24 h of admission: MP-DNA detected by MP polymerase chain reaction (PCR) tests by nasopharyngeal swab, phlegm respiratory pathogen eight (influenza A and influenza B, respiratory syncytial virus, adenovirus, metapneumovirus, parainfluenza virus type 1, 2, 3), blood respiratory pathogen IgM antibody nine (eosinophilic lung Legionella bacteria, *Mycoplasma pneumoniae*, Chlamydia, Rickettsia, adenovirus pneumonia, respiratory syncytial virus, influenza A virus, influenza B virus, and parainfluenza), tuberculosis and bacterial culture.

### Data collection

The clinical data of all children were collected as following: (1) basic information: name, gender, age, BMI, time to CTA/MRA (days), previous venous thrombo embolism (VTE), cardiovascular diseases (CVDs) and ICU admission. (2) clinical manifestations: Tmax, heart rate, main symptoms, distribution of embolism and outcome. (3) laboratory tests: routine blood tests, inflammatory markers, blood biochemistry, autoantibody, coagulation function and humoral immunity. (4) imaging examination results.

## Results

### Basic information

All five patients included in this study were between the ages of 5 and 9 years old, with a mean age of 6.8 years. Among these cases, three occurred in female patients. The body mass index (BMI) of the patients ranged from 17 to 30.7, with a mean BMI of 16.14. None of the patients had any history of family and basic disease, such as congenital heart disease, metabolic disease, thrombus, cardiovascular surgery, nephrotic syndrome and so on.

### Clinical features

All children in this study presented with persistent high fever and cough, accounting for 100% of the cases (*n* = 5). The maximum recorded temperature during the course of the disease ranged from 40.0 to 40.8 ℃. The duration of the illness before hospitalization at our department ranged from 6 to 12 days, with a mean duration of 8.6 days. Thrombosis occurs between the 13th and 22nd days after the onset of the disease. Notably, only one patient (case 4) experienced chest pain and hemoptysis. Conversely, the primary manifestations in children with cerebral embolism (case 5) included rapid loss of consciousness, limb paralysis, weakness, slurred speech, incomprehensible pronunciation, involuntary movement, and partial eyelid closure, among other symptoms. Unexpectedly, three cases (cases 1, 2, and 3) with pulmonary embolism were asymptomatic, comprising 60% of the cases (*n* = 3), as shown in Table 1. Additionally, damage to other organs was common, including the heart (*n* = 2, 40%), liver (*n* = 2, 40%), and kidneys (*n* = 5, 100%).

**Table 1:** The clinical features of pediatric MPP-associated embolism with PB

Clinical Features	Case1	Case2	Case3	Case4	Case5
Tmax (℃)	40	40.3	40.6	40.8	40
Time from onset	6	12	10	9	6
to admission (days)					
Admission (days)	14	12	14	31	28
Hemoptysis	No	No	No	Yes	No
Chest pain	No	No	No	Yes	No
Hypoxemia	Yes	Yes	Yes	Yes	Yes
Myocardial damage	No	Yes	No	Yes	No
Liver damage	No	Yes	No	Yes	No
Renal damage	Yes	Yes	Yes	Yes	Yes

### Laboratory examination

MP-DNA replication was detected in the alveolar lavage fluid of all five cases. MP antibodies were found in the blood of four children, with three of them having antibody titers reaching 1:640. The mean levels of D-dimer and fibrinogen (closest to CTA/MRA) were 9.2 ± 2.1 mg/L and 3.8 ± 1.2 g/L, respectively. The peripheral white blood cell (WBC) count of the five children was 12.9 ± 3.1 × 10^9^/L, and the mean neutrophil (N) percentage was 78.5%±7.8%. The mean platelet count was 350.8 ± 70.1 × 10^9^/L, and the average C-reactive protein (CRP) level was 76.3 ± 38.4 mg/L. The mean lactate dehydrogenase (LDH) level was 738.6 ± 122.9 U/L. Elevated erythrocyte sedimentation rate (ESR) levels were detected in four patients. The level of ferritin (FER) was 626.2 ± 396.8 ng/ml, with case 3 reaching as high as 1289 ng/ml, as shown in Table 2. The results of genetic testing for drug resistance in Mycoplasma pneumoniae revealed that cases 2 and 4 exhibited 2063/2064 + drug resistance mutations, while drug resistance testing was not conducted for the remaining three cases. Case 1’s antibody test showed a ANA( +) result with a titer of 1:320 and a granular-type karyotype. In contrast, case 4 tested negative for ACN antibodies. The antibody status of the remaining three cases was undetermined.

**Table 2:** The laboratory examination of pediatric MPP-associated embolism with PB

Laboratory Examination	Case1	Case2	Case3	Case4	Case5
D-dimer (closest to CTA/MRA)(mg/L)	7.2	> 5	11.92	7.1	10.7
D-dimer (hospital admission)(mg/L)	0.7	1.5	2.81	> 20	10.4
APTT (sec)	28.2	24.1	35.1	30.1	37.9
PT (sec)	12.3	10.9	13.5	15.6	12.5
Fg (closest to CTA/MRA) (g/L)	2.472	3.215	6.056	3.444	3.77
Fg (hospital admission) (g/L)	4.725	2.26	6.903	3.591	4.45
WBC (closest to CTA/MRA) (*10^9/L)	9	12.89	18.08	13.79	10.79
WBC (hospital admission) (*10^9/L)	18.09	17.17	15.81	14.01	7.49
NEUT%	78	82	89	78.6	64.94
PLT(*10^9/L)	328	464	248	374	340
CRP(mg/L)	47.1	35.5	79.05	146	74
PCT (ng/ml)	0.13	0.03	25.62	0.58	0.37
IL-6 (pg/ml)	5.59	15.21	247.4	27.67	14.55
LDH (U/L)	719	963	705	586	720
ALT (U/L)	15	68	17	56	16
IgM(g/L)	0.82	3.09	1.21	2.13	0.67
ESR (mm/hr)	27	52	/	30	34
FER (ng/ml)	147.8	705.9	1289	691.6	296.8

### Radiological examination and bronchoscopy

**Fig. 1:**
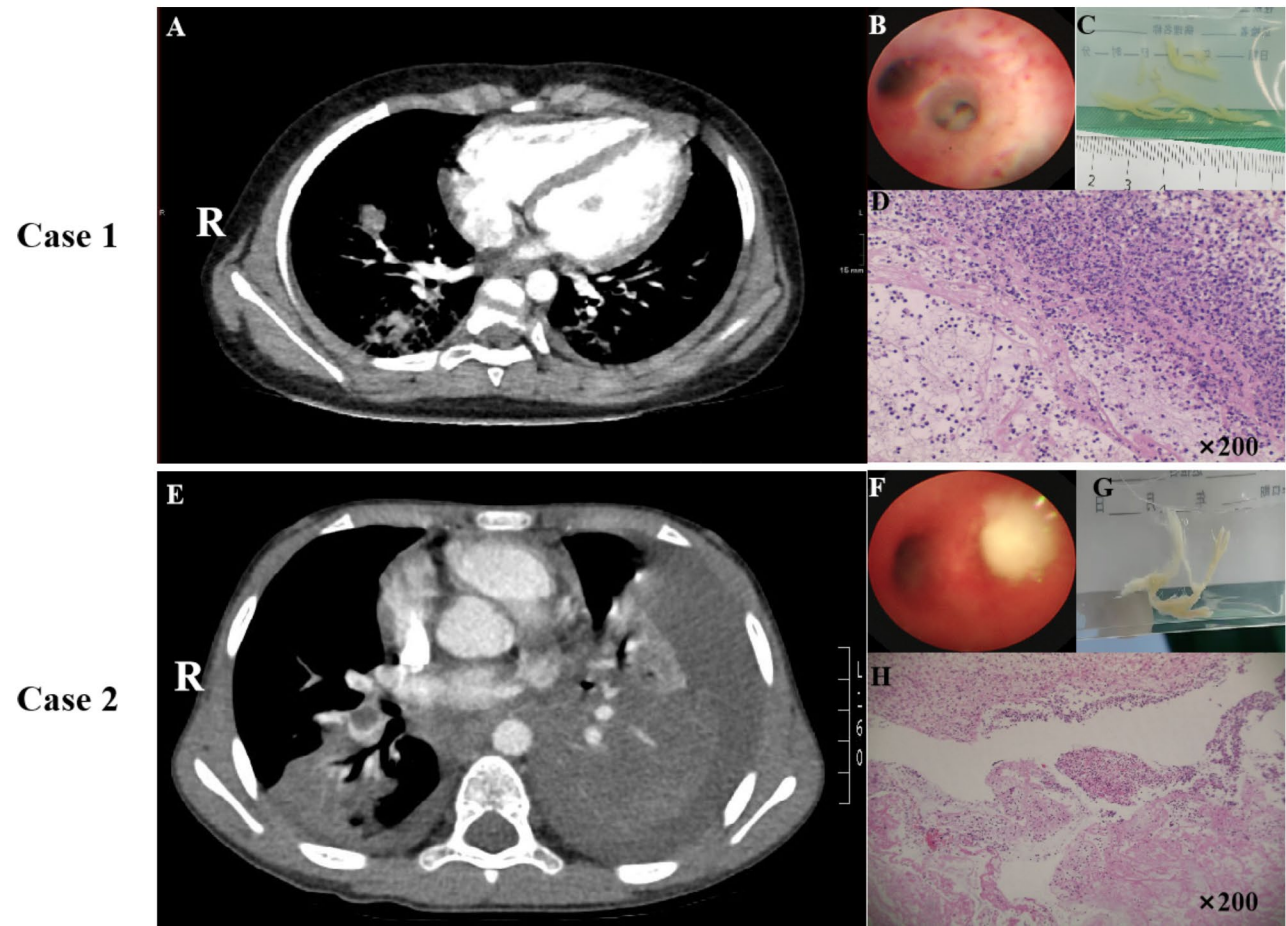
The examinations of MPP-associated thromboembolism with PB. (**A**) (Case 1) The chest CTA revealed filling defects in the bilateral inferior pulmonary arteries. (**B**) Bronchoscopy unveiled extensive cord-like plugs within the bronchial cavity of the anterior segment of the left upper lobe in Case 1. (**C**) Fiberoptic bronchoscopy extracted a plastic bronchial tree from the airway. (**D**) Pathological examination confirmed Type I plastic bronchitis in Case 1. (**E**) (Case 2) Chest CTA exhibited filling defects in the right lower pulmonary artery. (**F**) Bronchoscopy disclosed extensive cord-like plugs within the bronchial cavity of the anterior segment of the left upper lobe in Case 2. (**G**) Fiberoptic bronchoscopy removed a plastic bronchial tree from the airway of Case 2. (**H**) Pathological findings confirmed Type I plastic bronchitis in Case 2 through Hematoxylin and Eosin (H&E) staining

A pulmonary CT scan revealed pulmonary consolidation (≥ 2/3 lobe) in all cases (*n* = 5, 100%). However, atelectasis was present in 4 cases (*n* = 4, 80%), while mild to moderate pleural effusion and pleural thickening were observed in all cases (*n* = 5, 100%). Case 4 (*n* = 1, 20%) showed consolidation and necrosis. In the 4 children with pulmonary embolism (*n* = 4, 80%), the main thromboembolic vessels affected were the bilateral lower lobe pulmonary artery and right upper lobe pulmonary artery (as shown in Table 3). In the case of cerebral embolism (case 5), the primary embolic vessels were the left internal carotid artery and middle cerebral artery. Vascular malformations were not detected in any of the children based on imaging examinations. The average time from onset to bronchoscopy examination was 6–13 days (mean = 9.8 days). Bronchial casts were found in all cases, predominantly in the left lower lobe of the lung (Fig. 1).

**Table 3:** The radiological examination and bronchoscopy of pediatric MPP-associated embolism with PB

	Case1	Case2	Case3	Case4	Case5
Pulmonary consolidation	Yes	Yes	Yes	Yes	Yes
(⩾ 2/3 lobe)					
Pleural effusion	Yes	Yes	Yes	Yes	Yes
Atelectasis	Yes	Yes	Yes	No	Yes
Pleural thickening	Bilateral	Bilateral	Bilateral	Bilateral	Bilateral
Necrotizing pneumonia	No	No	No	Yes	No
Time from onset to	16	13	15	22	14
CTA/MRA (day)					
Embolic site	Bilateral inferior	Right lower	Right lower	Right lower	Left internal carotid artery、
	pulmonary arteries	pulmonary artery	pulmonary artery	pulmonary artery	Middle cerebral artery
Time from onset to	6	13	10	10	10
bronchoscopy (day)					
Number of bronchoscopy	3	3	3	3	2
Bronchial cast site	Right upper lobe	Left lower lobe	Left lower lobe	Left lower lobe	Both lung

### Treatment

All patients received empirical oral antibiotic treatment before admission. After admission, all five patients were administered azithromycin, methylprednisolone (2–10 mg/kg/d), low molecular weight heparin(2 IU/kg/d) for anticoagulation, and aspirin to inhibit platelet aggregation. As shown in, three patients (case2,3,5) also received gammaglobulin treatment. Additionally, the patient with cerebral infarction underwent treatment with a dehydrating agent to reduce intracranial pressure, while those with hepatic injury were administered hepatoprotective drugs. Finally, two patients (case2,5) with massive pleural effusion underwent chest thoracentesis.

### Outcome and follow-up

**Fig. 2:**
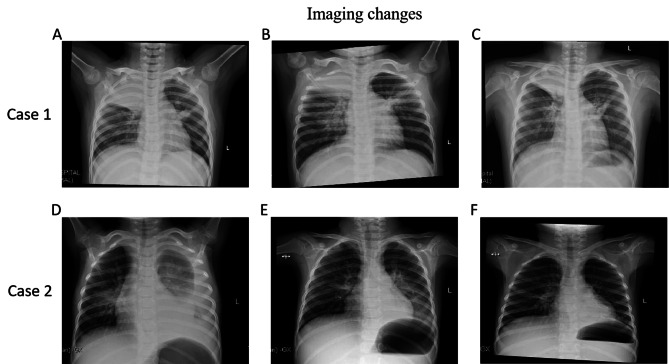
Imaging changes of case 1 and case 2. (**A**) Case 1: Chest radiography on day 3. (**B**) Chest radiography on day 6. (**C**) Chest radiography on day 10. (**D**) Case 2: Chest radiography on day 1. (**E**) Chest radiography on day 9. (**F**) Chest radiography on day 27

No fatalities were observed during the course of this study, and all cases presenting symptoms associated with thrombosis resolved, with laboratory indexes returning to within the normal range. Their chest X-ray findings approached or returned to normal (Fig. 2). In cases of pulmonary embolism, pulmonary computed tomography angiography (CTA) showed no filling defects after a period of 1–3 months. The child with cerebral embolism was followed up for a period of 12 months. While one of the patients experienced improved limb activity, they still suffered from sequelae including tremors and impaired motor function.

## Discussion

This study conducted a comprehensive search in authoritative databases including PubMed, Embase, and Web of Science by combining subject terms and free words. The subject terms comprised “*Mycoplasma Pneumoniae* Pneumonia,” “Thromboembolism,” and “Plastic Bronchitis.” Boolean operators “AND” and “OR” were employed to connect related synonyms and expanded vocabularies, such as “Mycoplasma infections” and “Pulmonary embolism,” ensuring the search results’ comprehensiveness and accuracy.

Previous reports have indicated that thrombosis or plastic bronchitis can manifest independently following *Mycoplasma pneumoniae* infection. Instances of severe *Mycoplasma pneumoniae* pneumonia accompanied by thrombosis and plastic bronchitis are not uncommon. Therefore, after excluding articles with incomplete data and cases of mixed infection, this paper focuses on eight relevant articles. Early detection and proactive interventions such as thrombolysis, anticoagulation, and bronchoscopy treatments often lead to improved prognoses shown in Table 4.

However, detailed case reports regarding thrombosis and plastic bronchitis resulting from *Mycoplasma pneumoniae* infection are lacking. Similarly, the mechanisms underlying *Mycoplasma pneumoniae*-induced thrombosis and plastic bronchitis remain unexplored. Although Liu J’s report mentioned four concurrent cases, no detailed introduction or summary was provided. This suggests that while cases of simultaneous thrombosis and plastic bronchitis may have occurred previously, they have not been thoroughly understood or documented. This paper presents five cases of concurrent thrombosis and plastic bronchitis, aiming to elucidate the underlying mechanisms and enhance understanding of these related conditions.

**Table 4:** The therapy regimens and outcome of the literature review of the MPP-associated thromboembolism with PB

References	Thromboembolism or PB	Cases in article	Study year	Age at the diagnosis	Sex	Duration from onset to discovery of thromboembolism or Pb (days)	Drug	Outcome
Chen L et al. [5]	Thromboembolism	49	January 2012 to December 2021	7.90 ± 2.72 years old	Male/Female ratio of 27:22	11.8(range, 4.0–24.0)	LMWH	Clinical conditions improved
Kim YS et al. [6]	Thromboembolism	4	September 2019 to February 2020	4–8 years old	NA	9.8(range, 6.0–14.0)	Methylprednisolone, Levofloxacin	Clinical conditions improved
Zhang T et al. [7]	Thromboembolism	1	30 July 2019	8 years old	Female	8	Latamoxef, Azithromycin, Methylprednisolone, Urokinase, LMWH, Aspirin	Clinical conditions improved
Liu J et al. [8]	Thromboembolism including 4 patients with plastic bronchitis	43	January 2013 to June 2019	7.92(range, 4.1–12.2) years old	Male/Female ratio of 23:20	7–31	Azithromycin, Moxifloxacin, Minocycline, LMWH, Methylprednisolone, Warfarin, Urokinase	Clinical conditions improved
Sheng CQ et al. [9]	Thromboembolism	7	January 2016 to August 2019	6–11(median, 8.0) years old	Male/Female ratio of 4:3	10–14	Macrolides, Third-generation Cephalosporins, carbapenems, Moxifloxacin, LMWH	Two patients succumbed to Acute Respiratory Distress Syndrome within 3 to 8 days following the surgical operation for pulmonary infarction. The remaining individuals achieved recovery.
Yang L et al. [10]	PB	133	February 2019 to January 2020 and August 2021 to July 2022	6.70 ± 2.49 years old	Male/Female ratio of 71:62	NA	NA	Clinical conditions improved
Zhang H et al. [11]	PB	52	January 2015 to December 2019	5.69(range, 4.89–6.48) years old	Male/Female ratio of 25/27	10.44 ± 2.94	Macrolide, Methylprednisolone	Six cases progressed to bronchiolitis obliterans, while the remaining individuals achieved recovery.
Zhang T et al. [12]	PB	3	March, 2016	1.9 years old	Female	9	Cephalothin, Azithromycin, Methylprednisolone	Recovery
			October, 2015	2.4 years old	Female	8	Cephalothin, Azithromycin, Methylprednisolone, Gamma globulin	Recovery
			April, 2016	4.3 years old	Male	7	Cephalothin, Azithromycin, Methylprednisolone, Gamma globulin	Recovery

**Fig. 3:**
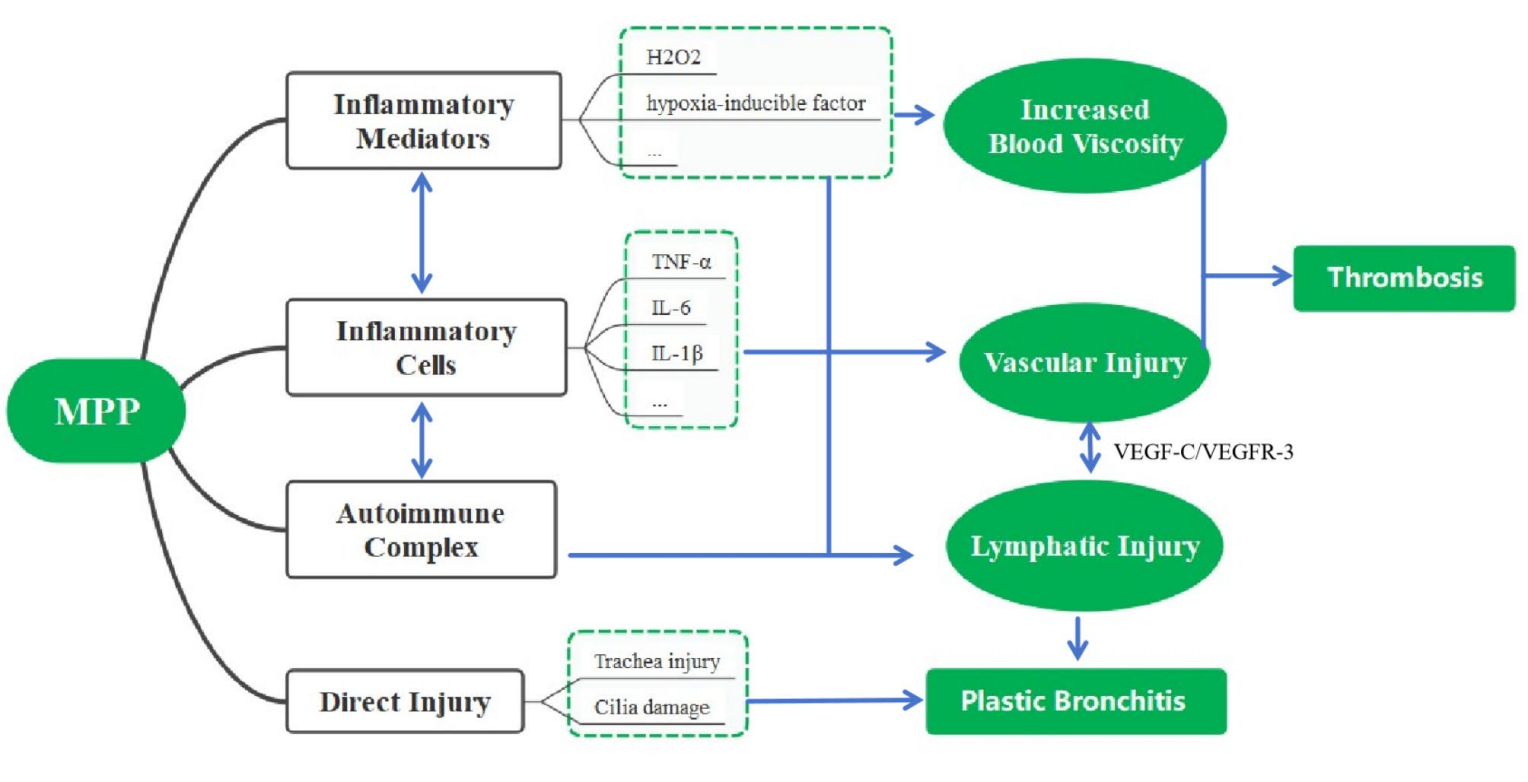
MPP-associated thromboembolism with PB mechanism diagram

*Mycoplasma pneumoniae* has been shown to directly or indirectly [3] induce airway inflammation by recruiting a substantial number of inflammatory cells and mediators, such as TNF-α, IL-1β, H_2_O_2_, and hypoxia-inducible factor [13]. These factors are known to lead to the structural damage to lymphatic vessels in the lungs [14]. Consequently, the disrupted structure of pulmonary lymphatic vessels compromises their ability to efficiently absorb inflammation [15] and regulate immune responses [16]. As a result, the accumulation of pleural effusion or pleurisy occurs [17], as observed in the cases presented in this study. Furthermore, *Mycoplasma pneumoniae* significantly stimulates the VEGF-C/VEGFR-3 signaling pathway, leading to lymphatic vessel hyperplasia [18]. This hyperplasia can establish a direct connection between the channels of pulmonary vein circulation. The leakage of lymph from damaged lymphatic vessels, along with the presence of inflammatory cells in the trachea, contributes to the development of type I plastic bronchitis, which is similar to the outcomes of Fontan surgery [19, 20]. Leaked lymph increases blood viscosity, damages blood vessel walls, and disrupts the balance of the fibrinolytic coagulation system, ultimately leading to the formation of blood clots. This may explain the higher susceptibility of pulmonary blood vessels to thromboembolism [21, 22], which is supported by the fact that four out of five thrombosis sites in our cases were observed in the lungs as shown in Fig. 3.

After an average time from onset of 6–13 days (mean = 9.8 days), imaging findings in patients demonstrated atelectasis and extensive pulmonary inflammation and consolidation. Bronchoscopy plays a crucial role in both diagnosing [23] and treating pulmonary inflammation and pulmonary ventilation dysfunction [24]. Following the criteria set by Wang L’s team, we conducted fiberoptic bronchoscopy and alveolar lavage fluid examination [25]. Fiberoptic bronchoscopy identified multiple plastic tracheal obstructions, which were successfully removed using forceps followed by alveolar lavage. Pathological analysis confirmed that the removed plastic substance was consistent with type I plastic bronchitis. Despite the alleviation of airway obstruction and identification of the pathogen, the consolidation area in the lungs did not show significant improvement on chest X-ray. Managing plastic bronchitis is a challenging task, often requiring repeated bronchoscopy for cast removal [26]. Subsequent bronchoscopy examination revealed a significant amount of mucus plugs in the trachea, which were cleared, resulting in improvement on chest X-ray.

After an average duration of 13–22 days from the onset of symptoms (mean = 16 days), the children’s blood oxygen saturation remained below normal levels. Given the elevation of D-dimer levels and the possibility of pulmonary embolism [27], the children underwent a CTA examination, which confirmed the presence of pulmonary embolism in 4 cases. In three of these cases, the emboli were located in the right lower pulmonary artery, while in 1 case, both lower pulmonary arteries were affected. It is noteworthy that only one case (case 4) presented with symptoms of chest pain and hemoptysis, while the remaining cases were asymptomatic. In case 5, typical symptoms of cerebral embolism were observed, including right hemiplegia, dysarthria, and cognitive impairment. Urgent MRA was performed, revealing interruption of blood flow in the left internal carotid artery and middle cerebral artery, thus confirming the diagnosis of cerebral embolism.

Hormonal therapy, in conjunction with appropriate antimicrobial agents, has demonstrated promising outcomes in the management of refractory MPP [1]. In our treatment regimen, azithromycin was included based on the literature indicating potential immunomodulatory and anti-inflammatory effects, even in the presence of drug resistance [28]. Notably, corticosteroids have been proven to effectively suppress inflammation and offer a cost-effective solution. In our previous investigations [29], early administration of high-dose corticosteroid pulse therapy for patients with refractory *Mycoplasma pneumoniae* pneumonia presenting with elevated laboratory inflammatory markers and pleural effusion was shown to be advantageous in controlling the patient’s condition and prognosis. Following hormone therapy in all five cases, a rapid interruption of the inflammatory storm and swift normalization of body temperature was observed within a few days. Subsequently, we gradually and steadily reduced the hormone dosage, and importantly, no recurrence of the patient’s condition was observed thereafter. These findings suggest that a more cautious approach to the utilization of corticosteroid hormones may be warranted for similar patients.

Currently, the treatment of thrombosis consists of anticoagulant therapy, thrombolytic therapy, and surgical thrombectomy [30]. However, the management of MPP complicated with thrombosis is relatively rare and lacks a standardized approach. Drawing from the American Guidelines for the Treatment of Thrombosis in Children [31] and our team’s previous experience in managing MPP thrombosis [7], we devised a conservative management protocol that involves using low molecular weight heparin (LMWH) for anticoagulation at a dosage of 2 IU/kg/day while regularly monitoring levels of D-dimer and fibrinogen (Fg). It is important to note that relying solely on D-dimer measurement to exclude pulmonary embolism may lead to the oversight of smaller subsegmental emboli [32]. Therefore, we incorporated Fg as an additional marker to more accurately assess the body’s coagulation status. LMWH has demonstrated effectiveness, safety, and good tolerability in children, thereby reducing the risk of pulmonary necrosis [33]. However, in cases where patients present with cardiovascular or cerebrovascular embolism or experience hemodynamic changes, we employ thrombolytic therapy (case 5).

Nonetheless, it is regrettable that several limitations persist, notably a restricted sample size. Moreover, there are instances of incomplete clinical data and laboratory examinations for patients. Thirdly, it is imperative to conduct further exploration into the specific mechanisms responsible for complications related to thrombosis and pulmonary embolism induced by MPP.

## Conclusion

Thromboembolism with plastic bronchitis associated with *Mycoplasma pneumoniae* pneumonia is a rare but potentially serious complication in children. Prompt recognition and management are crucial for improving patient outcomes. This case series highlights the diverse clinical presentations, diagnostic challenges, and treatment strategies for this unique clinical entity. Further research is needed to better understand the pathogenesis and optimal management of this condition.

## Data Availability

The datasets generated and/or analyzed during the current study are available from our manuscript.

## Abbreviations

LMWH: Low molecular weight heparin
MPP: Mycoplasma pneumoniae pneumonia
PB: Plastic bronchitis
